# Heterogeneities in Consumer Diet Quality and Health Outcomes of Consumers by Store Choice and Income

**DOI:** 10.3390/nu13041046

**Published:** 2021-03-24

**Authors:** Chandra K. Dhakal, Savin Khadka

**Affiliations:** Department of Agricultural and Applied Economics, University of Georgia, Athens, GA 30602, USA; sk56027@uga.edu

**Keywords:** food store choice, diet quality, obesity, body mass index, heterogeneities, FoodAPS

## Abstract

Obesity and other diet-related health conditions have received much attention in the public health literature over the past two decades. This study investigates the relationship between household food budget shares at different food outlets with diet quality and weight-related health outcomes in the United States. Our analysis used event-level food purchase data from the national household food acquisition and purchases survey (FoodAPS). We find that, after controlling for observables, food purchase location is significantly associated with diet quality and body mass index (BMI). Our findings indicate that larger food budget shares at convenience stores and restaurants are linked with poor diet quality based on the healthy eating index-2015 (HEI-2015) scores and higher BMI. We further explored potential heterogeneity on outcomes of interest across income groups. Results suggest heterogeneous effects may exist across income groups: low-income households, who spent a larger share of their food budget at convenience stores and fast-food restaurants are related to poor diet quality and more likely to be obese. Our findings will help improve understanding of the causes of diet-related health problems and may illuminate potential avenues of intervention to address obesity.

## 1. Introduction

Obesity and other diet-related health conditions have received much focus in the public health literature over the past two decades, and for a good reason. According to reports from the Centers for Disease Control and Prevention, in the year 2020, 43 percent of the US adult population over 20 years of age were obese, while more than two-thirds were overweight [1,2]. In comparison, only 23 percent of US adults were obese in 1988. This high incidence of obesity poses several challenges to both the US healthcare system as well as the economy. Tsai et al. [3] report that the medical costs of dealing with obesity-related issues account for up to 10 percent of US healthcare spending. Individuals dealing with obesity also spend 76 percent more on healthcare, on average, compared to individuals with a recommended body mass index (BMI) [4]. Further, obesity is also associated with an increased likelihood of cardiovascular diseases, type 2 diabetes, respiratory problems, depression, and stroke [5,6,7,8]. As such, improving our understanding of the factors that contribute to obesity and overweight is of prime importance.

While obesity is a result of numerous contributing factors that are difficult to identify exactly [9], research in this area has emphasized the retail food environment that is known to influence diet quality and overall health [10,11,12,13,14,15,16,17]. Existing studies in this area have focused mainly on supermarkets that offer healthful foods like fresh fruit and vegetables [18,19], suggesting that better access to a supermarket is associated with a reduced risk of obesity. Conversely, proximity to fast-food restaurants (i.e., those that provide only minimal table services and offer energy-dense food options) and convenience stores is related to an increased prevalence of obesity [20,21,22].

While vital, access to food outlets that carry relatively healthful foods is not the only factor that affects dietary quality and health. On the contrary, multiple studies have found that geographic variation in supermarket or supercenter proximity alone cannot account for most variation in a household’s primary food outlet and dietary quality [23,24]. These findings suggest that while access to food stores may have moderate influences on food purchase decisions, other household-level factors and socioeconomic aspects may have a larger effect. For instance, Allcott et al. [25] observe changes in the quality of household food purchases after changes in the retail environment and find that the healthfulness of households is only minimally responsive to changes in store proximity. Their study confirms the existence of a substantial nutrition-income relationship wherein households in the top income quartile buy groceries that are 0.56 standard deviations more healthful than the bottom income quartile, based on their version of the healthy eating index (HEI). Moreover, there is evidence that the choice of a store during food purchases impacts the dietary quality and health outcomes differentially across the socioeconomic strata [26,27,28,29].

A general assumption made in past studies investigating the relationship between local food environment food purchases and health outcomes is that individuals who shop at supermarkets and other large retail stores have a better nutrient profile of food purchases [15,30,31]. Arguments made in support of this notion are that supermarkets and wholesale stores sell a greater variety of foods with higher nutritional quality [32]. However, studies that rely on this notion suffer from several problems. First, several studies have only considered the customer’s physical proximity to the stores, perhaps due to data limitations [33,34]. Regarding investigating links between food purchases and obesity, a more useful metric would be to look at where individuals are buying their food instead of what store is the closest. Second, previous studies have usually considered food choices related to either food-at-home (FAH) [23,30,35,36] or food-away-from-home (FAFH) [26,37,38,39,40], while not many studies feature both [15,41] are being exceptions. Obviously, with regards to diet quality and subsequent health outcomes, we need to analyze both FAH and FAFH events for a more precise understanding. Third, these studies tend to emphasize the primary store type [31,42,43] or store in the neighborhood of residence [44,45] rather than food budget shares of expenditure at different store types [30,40,46]. Moreover, most of these studies do not consider all possible stores where people shop for food [41]. While families may spend a large share of their food expenditure at a supermarket, they may also rely on small retailers and convenience stores for their food-at-home purchases [47,48]. Exploring outcomes associated with different levels of budget shares at different outlet types may reveal the true relationship between consumers’ food store choices and their diet quality and health outcomes. Fourth, individual-level data linking nutritional values of grocery purchases and health outcomes to food purchase locations are scarce. Additionally, most studies looking at the relationship between food store choice and diet quality or weight-related health outcomes have mostly used limited data from a specific geographic location due to lack of nationally representative individual-level food purchase data [28,49,50]. We employ a unique and nationally representative dataset to establish a link between where households shop for food with the nutritional quality of purchases across income strata to address existing knowledge gaps.

This study builds upon a body of literature that establishes the association of the food store choices with food purchase quality and health outcomes. Specifically, we examine four important questions. First, are food dollar shares at different store types associated with diet quality? Second, are higher budget shares at convenience stores and fast-food restaurants associated with an increase in BMI levels? Third, are food budget share at different store types correlated with the incidence of obesity? Fourth, are there any heterogeneous effects in the nutritional quality and health outcomes across the income levels? Answering these questions will help to further our understanding of the sources of obesity and other weight-related issues and may illuminate potential avenues of intervention to curb the prevalence of obesity in the US.

## 2. Methodology

To empirically identify a relationship between nutritional quality, BMI, and the risk of obesity with a households’ choice of food establishments, we used the public-use version of the United States Department of Agriculture (USDA) national household food acquisition and purchases survey (FoodAPS). The unique FoodAPS datasets contain detailed information about the food purchased or otherwise acquired for consumption at home and away from home during a seven-day period. The survey consists of a nationally representative sample from 4826 households [51]. During the survey, each household provided details on the food acquisitions of its members over the past seven-day period. In addition, the primary respondent from each household also participated in two in-person interviews and up to three telephone interviews. Several previous studies have used the FoodAPS datasets to investigate food purchase behavior [43,52]. The FoodAPS datasets also provide information on sociodemographic characteristics, income level, BMI, and food acquisition (For more information on FoodAPS survey design and data collection process, see [51]). In addition to the personal interviews, households were instructed to scan barcodes on food, save receipts from food outlets, and record information in given food books. Altogether, there is information on 15,999 FAH acquisition events and 38,869 FAFH acquisition events in the FoodAPS data. This study considers both FAH and FAFH purchases while investigating the nutrition quality of food purchases quality, BMI, and obesity.

To measure the nutritional quality of food purchases, we used the composite HEI-2015 scores of all food items purchased during FAH and FAFH events. The HEI was initially developed in 1995 by the National Cancer Institute and the USDA to evaluate the healthfulness of American diets. HEI-2015 is the most current version of the HEI, which aligns with the 2015–2020 Dietary Guidelines for Americans [53]. HEI-2015 contains 13 components with nine adequacy components scores (whole fruit, total fruits, greens and beans, total vegetables, whole grains, total protein foods, dairy, seafood and plant proteins, and fatty acids) and four moderation component scores (saturated fats, sodium, added sugar, and refined grains). Each component score is based on a density basis out of 1000 calories except fatty acids, which is a ratio of unsaturated to saturated fatty acids. The total HEI-2015 score ranges from 0 to 100. The score increases with relative increases in the adequacy component scores and decreases when the moderation component scores. Higher scores indicate better diet quality [54]. Following Mancino et al. [55], we calculated HEI-2015 scores for both FAH and FAFH acquisitions. In our analysis, we used primary shopper’s information on self-reported BMI provided in the FoodAPS data (Individuals were asked to record their height and weight during the survey self-reported height and weight was used to compute the BMI). BMI is a continuous measure of body weight defined as weight in kilograms divided by the square of height in meters. We defined obesity as having a BMI of at least 30 [56]. The obesity indicator variable takes a value of 1 if the BMI of the respondent is at least 30 and 0 otherwise.

FoodAPS also has information about where households actually shop for food and how much they spend on each food purchase/acquire event. Based on types of stores household visited, we classified outlets into eight mutually exclusive: (1) supercenters, (2) supermarkets, (3) club or wholesale stores, (4) grocery stores, (5) convenience stores (Dollar stores, gas stations, and corner stores), (6) fast-food restaurants, (7) full-service restaurant, and (8) others (other category includes meals places of worship and school, clubs, food pantries, and dollar stores, and direct-to-consumer outlets. See [43,44] for a description of food store categories).

## 3. Statistical Analysis

Descriptive statistics were calculated to illustrate consumer food outlet choices and covariates in the sample. We used chi-squared tests to test the difference in covariates between low, medium, and high-income households, where a two-sided alpha level of 0.05 indicates statistical significance unless otherwise stated. The chi-squared test also compares the difference between food budget share across the food outlets. To explore the association of food budget share on each store type with nutritional quality and BMI, we used a multivariate ordinary least squares (OLS) regression. We regressed HEI-2015 scores and BMI on the food expenditure shares for different store types and covariates. Since obesity is a binary variable, we used a binary probit model to estimate the probability of being obese. FoodAPS data also contains information on household-level annual income as reported by the primary respondent (FoodAPS data provides information on income for all individuals over 16 years of age for six income categories: earnings; unemployment insurance; retirement and disability; welfare, child support, and alimony; investments; and other income sources. Household-level annual income is the sum of individual household members’ income). We divided households into three income groups: (a) incomes below 100% of the federal poverty line (FPL); (b) incomes between 100 and 185% of FPL; and (c) incomes at or above 185% of FPL, following Taylor and Villas-Boas [52]. We estimated four separate multivariate regressions for each response variable, one for the full sample and one for each income group. Regression models were adjusted for covariates to control for observed household characteristics. Beta coefficients and robust standard errors clustered at the household level are reported. We specified the covariates based on the store choice, diet quality, and obesity literature [35,57]. Previous studies have shown that socioeconomic and individual factors are strong predictors of diet quality and obesity [35,58]. Based on these studies, we included household size, children, older adults, Hispanic race, Asian, Black, Non-white, obese members, smoker, distance to primary stores, college education, age, gender, and marital status as control variables. We also confirmed the validity of basic OLS assumptions, including the normality of residuals. The University of Georgia Institutional Review Board (IRB) determined that the proposed study is not research involving human subjects. Data were analyzed using STATA version 14 (StataCorp LLC, College Station, TX, USA) and accounted for survey design and sample weights.

## 4. Results

Table 1 reports summary statistics of outcome variables across different types of food establishments in our sample of 4316 families over a seven-day period. We observed that the vast majority shopped for food at either supercenters or supermarkets. Our sample showed that HEI-2015 scores differ based on the type of establishment visited for food purchases. Further, the incidence of obesity also varied according to the establishments visited for food purchases. At the same time, there were no differences in BMI outcomes observed for families based on their purchase locations. Findings suggest that expenditure shares are different across different store types (*p*-value for both <0.001), with supercenters accounting for the highest expenditure shares among respondents.

Table 2 reports summary statistics (mean and standard errors clustered at the household-level) for weekly household expenditures across different food establishments based on sociodemographic and household characteristics in our sample. Weekly household expenditures at wholesale locations were high for large families and families with members older than average. Among minority groups, black families were more likely to shop at convenience stores and fast-food outlets, whereas Hispanic families acquired most of their food supplies from grocery stores. Families of Asian origin, on the other hand, preferred to shop for food at wholesale stores. Results indicate that expenditure at stores also depends on the presence of a child in the family, ownership of a personal vehicle, education, and marital status. In rural areas, food expenditure was highest at convenience stores, while families with access to personal vehicles were more likely to purchase foods at wholesale locations.

Table 3 shows the estimates for HEI-2015 based on expenditure at different types of food establishments as well as sociodemographic and household characteristics. Columns 3, 4, and 5 provide HEI- 2015 estimates for samples restricted to households that are below the FPL, between 100 to 185% of the FPL, and above 185% of the FPL, respectively. Based on our full-specification in Column 2, we found that larger food budget share at club or wholesale stores is associated with higher HEI- 2015 scores relative to supermarkets. This effect was statistically significant (*p*-value < 0.01) and was the highest in families that earned more than 185% of the FPL.

Conversely, shopping for food at convenience stores and fast-food restaurants was linked with reductions in diet quality. Our estimates suggest relying on convenience stores and fast-food restaurants for food is correlated with lower HEI-2015 scores (*p*-value < 0.01). Similarly, in families where full-service restaurants accounted for the largest share in food expenditure, HEI-2015 scores were lower by 6.46 points (*p*-value < 0.01). For families below the FPL, we found that poor-quality diets were related to higher food budget expenditure at convenience stores and fast-food restaurants, where the effect was a decrease in HEI-2015 scores by 3.47 (*p*-value < 0.1) and 7.75 points (*p*-value < 0.01), respectively. In contrast, a larger budget share at club or wholesale stores for the same group was positively and significantly associated with better diet quality (*p*-value < 0.01). Among families earning more than 185% of the FPL, on the other hand, low-quality diets were associated with higher spending at convenience stores, fast-food restaurants, and full-service restaurants. Thus, our main finding regarding diet quality is that families should be encouraged to shop for food at the club or wholesale stores over convenience stores and restaurants.

Table 3 also reveals associations between living in rural areas and lower diet quality. Our full sample regression found that households with tobacco users purchased foods that scored lower on dietary quality, particularly among low-income families. We also observed significant differences in diet quality across races, where being Asian was significantly correlated with better diet quality.

Table 4 provides estimates from multivariate OLS regressions with BMI levels as the dependent variable. Based on our results, having higher food budget shares at convenience stores, fast-food, or full-service restaurants was significantly linked with higher BMI levels. The coefficient on club or wholesale stores shows that higher expenditure at those outlets was negatively linked with BMI for the low-income subsample (*p*-value < 0.1). For the subsample of households with income between 100 to 185% of the FPL, increased food dollar share at convenience stores and fast-food restaurants were associated with a higher BMI (*p*-value < 0.05). However, for the high-income subsample (Income >185% FPL), larger expenditure share at grocery stores and full-service restaurants were correlated with higher BMI (*p*-value < 0.05).

Sociodemographic and individual characteristics have mixed results depending on the income level. Being Asian was negatively associated with BMI in our full sample, with the most significant effect on low-income families. Our findings suggest that the presence of young children in the household is associated with a slight decrease in BMI levels. Education also displays an inverse relationship with BMI levels, with the most substantial effect observed in households with college graduates.

Table 5 provides estimates for the binary probit model with the incidence of obesity as the dependent variable. Results are also provided separately for FPL-based income groups (Columns 2, 3, and 4). Our analysis showed that the incidence of obesity is not connected with the primary food purchase location. However, some statistically significant effects were observed across FPL income groups. For instance, a larger food budget share at fast-food establishments was positively related to obesity for low-income households (below 185% of FPL, *p*-value < 0.05). Our findings also suggest that race was associated with obesity. In our full sample, we found that Hispanics were more likely to be obese, while Asian families were less likely to be obese than white families. Moreover, the incidence of obesity was also higher in rural areas for high-income households (*p*-value < 0.1). Similarly, a family with a child under six years of age was negatively associated with the likelihood of obesity (*p*-value < 0.01).

## 5. Discussion

This study investigates the relationship between household food outlet choice and (1) diet quality and (2) weight-related health outcomes (BMI and obesity), using FoodAPS data while accounting for several key issues. Our findings indicated that household food expenditure at different food retail outlets was associated with their diet quality and health outcomes—larger food budget shares at convenience stores and fast-food restaurants were linked to poor diet quality and higher BMI levels. Further, this effect was concentrated on low-income households.

Several studies have investigated the association between retail food outlets with diet quality to understand the risk factors associated with obesity [35,57]. These studies indicate that the diet quality of food purchases and incidence of obesity varies with households’ choice of food stores. While our study was not designed to establish causative relationships, our findings support existing reports in the public health literature that explore the effects of where people shop for food on diet quality and weighted-related health outcomes (For details about causality between food environment and nutrition, please see Zhen [59]. He has reviewed the studies that attempt to establish the causal relationship between food access and nutrition/health). The contribution of FAFH, particularly fast-food, in increasing caloric intake and reducing diet quality are well-documented [20,55,60]. In the case of convenience stores, the reduction in diet quality probably arises from the prevalence of processed and energy-dense foods [33]. These foods generally lack fiber, vitamins, minerals, and phytonutrients, while containing many ingredients that reduce healthfulness, such as fillers, preservatives, hydrogenated or saturated fats, sodium, artificial colors, and flavors [61]. In contrast, wholesale stores and supercenters offer a wider variety of foods, including an abundance of those that promote good health, such as whole grains, fruits, vegetables, and bulk produce. Thomsen et al. [19] argue this may be the reason why households spending more at relatively larger stores may be more likely to buy healthful food and have a better diet.

Many prior studies have investigated the relationship between the primary location of food purchases and BMI, with no clear consensus. While some evidence for a positive relationship has been reported [62,63,64], numerous contradicting reports deny the existence of such a relationship [35,65,66,67]. However, our study differs from most of these, including the reports cited here, along one critical dimension: we used household food dollar shares at different store types instead of using the primary or the closest shopping store. Evidence already indicates that using the closest grocery store in terms of distance to study health outcomes is flawed [31,68,69]) as households usually do not shop at the grocery store that is closest to them [70]. Based on actual food budget expenditure data, our results revealed that BMI links with the type of store utilized for grocery shopping after controlling for sociodemographic and household characteristics. In our sample, the shoppers who spent the largest share of their food dollars at a convenience store had a BMI that was 3.03 points higher than the supermarket expenditure share. Similarly, households that used full-service restaurants as their primary source of food supply also had a BMI that was 2.5 points higher than supermarket spending share. The effects of FAFH on BMI were mainly strong on households with the highest annual incomes, which is consistent with established results about the impact of income on food expenditure away from home [40,71]. Our findings are also in agreement with Park et al. [72], who used the Nationwide Food Consumption Survey to estimate elasticities for twelve food commodity groups and found that, while own-price elasticities were similar between income groups for most commodities, income elasticities were consistently higher for the lower-income group, suggesting that income is an important determinant of food purchase decisions and diet quality.

While the current consensus is that the type of store used as the primary source of the food supply is linked to the prevalence of obesity, our analysis did not find any links in the full sample. This may be due to two reasons. First, as previously stated, our study differs from most existing ones in using shares of expenditure instead of physical distance as the basis of classification. While there are numerous reports of a positive link between obesity and the retail food environment [73,74], most of them are based on proximity to store. The literature on the role of spending behavior on obesity is growing, and some early contributions have been provided by Chen et al. [57,75], which our paper adds to. Second, several empirical papers have also reported a lack of relationship between retail food environment and risk of obesity. For instance, Ghosh-Dastidar et al. [76] find that, while both distances to store and food prices were positively associated with obesity, only prices were significant when the two were jointly modeled, suggesting that any relationship, if it does exist, is perhaps weak. This is also supported by the findings of Chen et al. [57]. They investigated the impact of the retail food environment on the risk of obesity and found that the food environment at the neighborhood level had a smaller effect on obesity and overweight than individual or household level characteristics. However, a positive and significant association between food budget share at fast-food restaurants and obesity among low-income neighborhoods corroborates most previous research [77]. Possible mechanisms to explain the positive relation between fast-food restaurant expenditure shares and obesity, particularly among the low-income group, include higher access to a fast-food restaurant in the low-income neighborhood [78], low food and nutrition knowledge [79], lack of cooking equipment, and fewer cooking skills [80].

Household, socioeconomic, and individual characteristics are known to influence food purchasing behaviors, diet quality, and health outcomes [58]. Our finding that college education attainment is positively and significantly associated with better diet quality is also consistent with past studies showing that socioeconomic status (income and education level) accounts for a greater share of variation in diet quality than store types [81,82]. Moreover, the differential impact of race on diet quality and health outcomes, such as obesity, is also established, with research showing that Asians have overall more healthful diets and a lower likelihood of being obese than non-Hispanic white and non-Hispanic black adults [2]. Tobacco use was also linked with overall diet quality and obesity. Studies on the association between diet quality and tobacco consumption indicate that tobacco users have less healthful diets [83] and a higher prevalence of obesity [84]. Further, individuals with lower education have been reported to consume less healthful foods and positively correlated with higher body weight [85]. We also found that having a college degree is associated with lower body weight and lower risk of obesity, supporting a previous report by Burgoine et al. [26].

## 6. Limitations

This study contributes to the local food retail environment and obesity literature by accounting for several issues mentioned in the previous section. However, results from our analysis have some caveats and should be interpreted with caution. We utilized the most recent nationally representative cross-sectional data collected between April 2012 and January 2013 to represent the food items purchased and acquired over seven-day periods, which might not entirely reflect long-run household food purchase behaviors. Besides, our results are based on food purchases and acquisitions, which might not precisely measure food intake due to food storage, waste, and intrahousehold sharing [86]. Further, due to the cross-sectional nature of the data, our results only reveal associations between the households’ food store choices and diet-related outcomes, and this study does not aim to establish any causal relationships between the variable of interest. We relied on self-reported data on sociodemographics and other household characteristics, which may be subject to misspecification and memory bias. The FoodAPS data also does not include other variables, for example, physical activities and food consumption behaviors, which are important predictors of obesity.

## 7. Conclusions

Food availability and accessibility have been well studied in relation to nutritional quality and health outcomes. Where households shop for food and how much they spend at different food outlets are extremely important questions in determining diet quality and risk of obesity. Our study examined the association between expenditure shares at retail outlets and the healthfulness of food purchase and health outcomes using a detailed and nationally representative large data set. We report four main findings. First, food dollar shares at different outlets are associated with diet quality. Second, larger food dollars at convenience stores and fast-food restaurants are also linked to increased BMI levels. Third, we found no connections between food spending at stores and the prevalence of obesity in our full sample. Finally, we provide evidence of heterogeneity on all three outcomes based on income levels. Low-income households, who spent a larger share of their food budget at convenience stores and fast-food restaurants, were associated with lower diet quality and were more likely to be obese.

## Figures and Tables

**Table 1:** Summary statistics of outcome variables by store choices in the sample.

Variable	Full Sample		Store Types							*p*-Value
		Supercenter	Supermarket	Club/Wholesale Store	Grocery Store	Convenience Store	FFR	FSR	Other	
	(1)	(2)	(3)	(4)	(5)	(6)	(7)	(8)	(9)	(10)
HEI-2015	49.09	49.45	50.29	57.42	47.21	40.29	44.44	47.93	47.76	<0.001
	(0.34)	(0.63)	(0.54)	(2.00)	(1.71)	(1.61)	(1.01)	(0.82)	(1.25)	
BMI	27.47	27.58	27.03	26.42	27.97	28.09	28.46	27.90	26.68	0.0023
	(0.17)	(0.35)	(0.32)	(0.57)	(0.67)	(0.80)	(0.57)	(0.38)	(0.70)	
Obese (1/0)	0.32	0.36	0.29	0.22	0.33	0.31	0.39	0.33	0.23	<0.001
	(0.01)	(0.02)	(0.02)	(0.04)	(0.06)	(0.06)	(0.04)	(0.03)	(0.04)	
N	4316	1517	1188	175	144	161	348	577	206	

The estimates use sample weights and control for survey design. Robust standard errors clustered at the household level in parentheses. HEI-2015: healthy eating index-2015; BMI: body mass index; FFR: fast-food restaurant; FSR: full-service restaurant.

**Table 2:** Summary statistics of covariates by store types in the sample.

Variable	Full Sample	Store Types							
		Supercenter	Supermarket	Club/wholesale Store	Grocery Store	Convenience Store	FFR	FSR	Other
	(1)	(2)	(3)	(4)	(5)	(6)	(7)	(8)	(9)
Household size (log)	0.72	0.72	0.74	0.90	0.65	0.52	0.70	0.69	0.65
	(0.01)	(0.03)	(0.03)	(0.07)	(0.08)	(0.07)	(0.05)	(0.04)	(0.06)
Hispanic (1/0)	0.13	0.14	0.12	0.12	0.17	0.07	0.13	0.15	0.10
	(0.01)	(0.01)	(0.01)	(0.03)	(0.04)	(0.03)	(0.02)	(0.02)	(0.02)
Black (1/0)	0.12	0.09	0.11	0.06	0.10	0.20	0.19	0.16	0.14
	(0.01)	(0.01)	(0.01)	(0.02)	(0.05)	(0.05)	(0.03)	(0.02)	(0.03)
Asian (1/0)	0.04	0.03	0.03	0.11	0.04	0.01	0.02	0.08	0.02
	(0.00)	(0.01)	(0.01)	(0.03)	(0.02)	(0.00)	(0.01)	(0.02)	(0.01)
Child under the age of six (1/0)	0.15	0.16	0.15	0.19	0.21	0.15	0.13	0.11	0.10
	(0.01)	(0.01)	(0.01)	(0.04)	(0.05)	(0.04)	(0.02)	(0.02)	(0.03)
Rural (1/0)	0.34	0.42	0.29	0.20	0.39	0.54	0.25	0.29	0.43
	(0.01)	(0.02)	(0.02)	(0.05)	(0.06)	(0.07)	(0.04)	(0.03)	(0.05)
Any vehicle (1/0)	0.90	0.89	0.90	0.98	0.86	0.68	0.87	0.92	0.91
	(0.01)	(0.01)	(0.01)	(0.01)	(0.04)	(0.06)	(0.02)	(0.01)	(0.02)
Tobacco (1/0)	0.19	0.19	0.18	0.08	0.26	0.56	0.28	0.14	0.23
	(0.01)	(0.02)	(0.02)	(0.02)	(0.05)	(0.07)	(0.03)	(0.02)	(0.05)
Age (years)	49.95	50.67	51.50	52.19	50.52	46.70	45.15	47.95	50.05
	(0.44)	(0.83)	(0.84)	(1.66)	(2.47)	(2.62)	(1.20)	(1.02)	(1.68)
No diploma (1/0)	0.24	0.29	0.24	0.12	0.31	0.31	0.26	0.16	0.22
	(0.01)	(0.02)	(0.02)	(0.03)	(0.06)	(0.06)	(0.03)	(0.02)	(0.05)
High school (1/0)	0.34	0.35	0.34	0.29	0.19	0.38	0.36	0.34	0.37
	(0.01)	(0.02)	(0.02)	(0.05)	(0.05)	(0.07)	(0.04)	(0.03)	(0.05)
College degree (1/0)	0.33	0.27	0.33	0.54	0.30	0.17	0.29	0.43	0.32
	(0.01)	(0.02)	(0.02)	(0.05)	(0.07)	(0.06)	(0.04)	(0.03)	(0.05)
Married (1/0)	0.45	0.46	0.47	0.67	0.39	0.20	0.29	0.45	0.46
	(0.01)	(0.02)	(0.02)	(0.06)	(0.06)	(0.05)	(0.04)	(0.03)	(0.05)
Male (1/0)	0.68	0.70	0.72	0.72	0.71	0.62	0.64	0.57	0.66
	(0.01)	(0.02)	(0.02)	(0.05)	(0.06)	(0.07)	(0.04)	(0.03)	(0.05)
Expenditure share		0.27	0.23	0.04	0.03	0.04	0.14	0.17	0.08
		(0.26)	(0.01)	(0.00)	(0.00)	(0.00)	(0.00)	(0.01)	(0.00)

The estimates use sample weights and control for survey design. Robust standard errors clustered at the household level in parentheses. FFR: fast-food restaurant; FSR: full-service restaurant.

**Table 3:** Association between healthy eating index 2015 and food outlet types by income.

Variable	Healthy Eating Index 2015 (0–100)			
	Full Model	Income < 100% FPL	Income 100−185% FPL	Income > 185% FPL
	(1)	(2)	(3)	(4)
Store type (ref: Supermarket)				
Supercenter (share)	−0.25	1.11	−3.92 *	0.44
	(1.19)	(2.20)	(2.33)	(1.55)
Club/wholesale store (share)	7.78 ***	1.98 ***	0.93	9.19 **
	(2.95)	(0.60)	(4.14)	(3.61)
Grocery store (share)	−2.31	−3.38	−3.42	−1.84
	(2.25)	(3.23)	(4.03)	(3.16)
Convenience (share)	−8.56 ***	−3.47 *	−7.94 *	−14.49 ***
	(2.30)	(1.53)	(4.10)	(3.84)
Fast food restaurant (share)	−8.58 ***	−7.75 ***	−7.14 *	−9.59 ***
	(1.88)	(2.92)	(4.03)	(2.39)
Full−service restaurant (share)	−6.46 ***	−6.79 *	−13.93 ***	−5.22 ***
	(1.57)	(3.73)	(3.00)	(1.94)
Other (share)	−3.63 *	−4.83	−11.07 ***	−1.76
	(1.87)	(3.48)	(4.08)	(2.39)
Household size (log)	−2.84 ***	−1.54	−3.11 **	−3.01 ***
	(0.62)	(1.12)	(1.24)	(0.82)
Race/ethnicity (ref: White)				
Hispanic (1/0)	1.39	4.48	1.92	1.22
	(1.88)	(4.61)	(2.19)	(2.65)
Black (1/0)	−1.65	0.08	1.46	−2.62
	(1.92)	(4.35)	(2.15)	(2.84)
Asian (1/0)	3.17 **	1.67	8.20 **	2.61
	(1.02)	(4.24)	(3.29)	(2.91)
Child under the age of six (1/0)	0.32	−1.02	−0.03	0.30
	(0.76)	(1.24)	(1.23)	(1.03)
Rural (1/0)	−0.90	−0.20	−0.08	−1.33
	(0.77)	(1.64)	(1.57)	(0.93)
Any vehicle (1/0)	1.38	0.69	1.83	0.01
	(0.98)	(1.33)	(1.80)	(1.67)
Tobacco (1/0)	−4.10 ***	−6.41 ***	−3.99 ***	−3.44 ***
	(0.79)	(1.51)	(1.46)	(1.12)
Age (years)	0.06 ***	0.01	0.04	0.07 ***
	(0.02)	(0.05)	(0.04)	(0.03)
Education level (ref: Non-diploma)				
High school (1/0)	2.29 **	0.43	1.05	2.49
	(0.96)	(1.54)	(1.98)	(1.57)
College degree (1/0)	5.64 ***	6.07 ***	0.12	5.57 ***
	(1.04)	(2.08)	(2.14)	(1.60)
Married (1/0)	2.91 ***	−1.14	3.03 **	2.81 ***
	(0.65)	(1.30)	(1.25)	(0.84)
Male (1/0)	0.57	0.30	−1.55	1.27
	(0.75)	(1.62)	(1.55)	(0.92)
Constant	46.57 ***	47.81 ***	49.82 ***	47.29 ***
	(2.66)	(5.50)	(4.59)	(3.77)
Observations	4316	1005	1224	2087

The estimates use sample weights and control for survey design. Robust standard errors clustered at the household level in parentheses. FPL: federal poverty line. * *p* < 0.1, ** *p* < 0.05, *** *p* < 0.01.

**Table 4:** Association between body mass index and food outlet types by income.

Variable	Body Mass Index (kg/m^2^)			
	Full Model	Income < 100% FPL	Income 100–185% FPL	Income > 185% FPL
	(1)	(2)	(3)	(4)
Store type (ref: Supermarket)				
Supercenter (share)	0.90	−0.35	1.66	0.92
	(0.72)	(1.28)	(1.06)	(0.97)
Club/wholesale store (share)	1.40	−3.99 *	2.04	2.03
	(1.14)	(2.21)	(2.39)	(1.37)
Grocery store (share)	1.37	−1.70	−1.74	3.47 **
	(1.16)	(2.48)	(1.43)	(1.59)
Convenience (share)	3.03 **	−0.46	5.00 **	2.83
	(1.39)	(2.35)	(2.35)	(1.94)
Fast-food restaurant (share)	1.48 ***	0.31 ***	2.08 **	1.92
	(0.45)	(0.07)	(0.87)	(1.22)
Full-service restaurant (share)	2.50 ***	−0.37	1.49	3.31 ***
	(0.90)	(1.88)	(2.13)	(1.08)
Other (share)	−0.70	−2.21	−0.39	−0.03
	(1.10)	(1.91)	(2.04)	(1.48)
Household size (log)	0.29	0.81	1.85 ***	−0.28
	(0.37)	(1.04)	(0.58)	(0.48)
Race/ethnicity (ref: White)				
Hispanic (1/0)	0.33	−3.09 **	−1.89	2.06
	(1.26)	(1.38)	(1.42)	(1.85)
Black (1/0)	2.05	−0.28	−0.08	3.45 *
	(1.27)	(1.45)	(1.41)	(1.87)
Asian (1/0)	−2.60 **	−6.62 ***	−2.76	−1.07
	(1.29)	(1.74)	(1.96)	(1.82)
Child under the age of six (1/0)	−0.72 **	−1.91	−2.81 ***	−0.26
	(0.30)	(1.37)	(0.95)	(0.63)
Rural (1/0)	0.48	0.69	0.83	0.51
	(0.42)	(1.13)	(0.83)	(0.51)
Any vehicle (1/0)	−0.02	−0.19	−0.96	1.40
	(0.52)	(0.77)	(0.98)	(0.90)
Tobacco (1/0)	−0.75	−1.04	−1.68	−0.78
	(0.53)	(0.81)	(0.89)	(0.78)
Age (years)	0.01	0.02	−0.01	0.02
	(0.01)	(0.03)	(0.02)	(0.02)
Education level (ref: Non-diploma)				
High school (1/0)	−0.65	−0.49	0.65	−1.30
	(0.63)	(1.18)	(1.12)	(0.96)
College degree (1/0)	−2.25 ***	−1.09	−1.71	−2.80 ***
	(0.63)	(1.30)	(1.17)	(0.94)
Married (1/0)	−0.17	−0.78	0.16	0.21
	(0.36)	(1.01)	(0.76)	(0.46)
Male (1/0)	−0.54	1.61 **	−0.50	−0.90 *
	(0.37)	(0.77)	(0.60)	(0.47)
Constant	27.42 ***	30.10 ***	29.61 ***	24.64 ***
	(1.68)	(2.72)	(2.35)	(2.33)
Observations	4316	1005	1224	2087

The estimates use sample weights and control for survey design. Robust standard errors clustered at the household level in parentheses. FPL: federal poverty line. * *p* < 0.1, ** *p* < 0.05, *** *p* < 0.01.

**Table 5:** Association of incidence of obesity and food budget share at different stores and covariates.

Variable	Obesity (1/0)			
	Full Model	Income < 100% FPL	Income 100–185% FPL	Income > 185% FPL
	(1)	(2)	(3)	(4)
Store type (ref: Supermarket)				
Supercenter (share)	−0.28	−0.39	−0.01	0.32
	(0.21)	(0.35)	(0.40)	(0.28)
Club/wholesale store (share)	−0.21	−0.51	−1.49	1.33
	(0.41)	(0.46)	(1.46)	(0.85)
Grocery store (share)	0.48	−0.06	0.83	0.97
	(0.49)	(0.12)	(1.43)	(0.99)
Convenience (share)	−0.06	0.38	2.54	−0.58
	(0.39)	(0.27)	(2.00)	(0.67)
Fast-food restaurant (share)	0.17	0.15 **	0.41 **	0.60
	(0.34)	(0.07)	(0.18)	(0.53)
Full-service restaurant (share)	−0.16	0.11	−0.96	0.14
	(0.27)	(0.68)	(0.76)	(0.38)
Other (share)	0.25	−0.25	0.03	0.75
	(0.39)	(0.67)	(1.04)	(0.64)
Household size (log)	−0.09	0.25	−0.32	−0.20
	(0.12)	(0.25)	(0.26)	(0.18)
Race/ethnicity (ref: White)				
Hispanic (1/0)	0.21 **	−3.54	−3.12	0.71 *
	(0.10)	(2.27)	(4.76)	(0.42)
Black (1/0)	0.05	−3.29	−4.09	0.55
	(0.33)	(2.27)	(4.76)	(0.44)
Asian (1/0)	−0.30 ***	0.00	0.00	−0.52
	(0.6)	(.00)	(.00)	(0.51)
Child under age 6 years (1/0)	−0.05 ***	−0.05	−0.17	0.15 **
	(0.01)	(0.30)	(0.27)	(0.06)
Rural (1/0)	0.22	0.21	0.04	0.32 *
	(0.13)	(0.33)	(0.28)	(0.19)
Any vehicle (1/0)	0.29 **	0.38	0.36	0.17
	(0.14)	(0.25)	(0.30)	(0.33)
Tobacco (1/0)	0.26 *	0.30	0.15	0.36
	(0.13)	(0.30)	(0.29)	(0.25)
Age (years)	0.01	−0.01	0.00	0.01
	(0.00)	(0.01)	(0.01)	(0.01)
Education level (ref: Non-diploma)				
High school (1/0)	0.24	0.43	0.45	−0.07
	(0.15)	(0.36)	(0.29)	(0.33)
College degree (1/0)	−0.27 **	0.47	−0.72 **	−0.04
	(0.11)	(0.51)	(0.27)	(0.34)
Married (1/0)	−0.27 **	−0.78 ***	−0.02	−0.21
	(0.11)	(0.27)	(0.25)	(0.17)
Male (1/0)	−0.86 ***	0.00	0.00	−0.66 **
	(0.23)	(.00)	(.00)	(0.26)
Constant	2.47 ***	5.06	5.73	1.93 ***
	(0.48)	(256.27)	(431.76)	(0.70)
Observations	4316	706	889	2087

The estimates use sample weights and control for survey design. Robust standard errors clustered at the household level in parentheses. FPL: federal poverty line. * *p* < 0.1, ** *p* < 0.05, *** *p* < 0.01.

## Data Availability

Publicly available datasets were analyzed in this study. This data can be found here: https://www.ers.usda.gov/data-products/foodaps-national-household-food-acquisition-and-purchase-survey/.

## Abbreviations

BMI	Body mass index
HEI	Healthy eating index
FoodAPS	Food acquisition and purchase survey
FPL	Federal poverty line
FAH	Food at home
FAFH	Food away from home
USDA	United States Department of Agriculture
OLS	Ordinary least squares
